# The delayed strengthening of synaptic connectivity in the amygdala depends on NMDA receptor activation during acute stress

**DOI:** 10.14814/phy2.13002

**Published:** 2016-10-26

**Authors:** Farhana Yasmin, Kapil Saxena, Bruce S. McEwen, Sumantra Chattarji

**Affiliations:** ^1^ National Centre for Biological SciencesBangaloreIndia; ^2^ Centre for Brain Development and RepairInstitute for Stem Cell Biology and Regenerative MedicineBangaloreIndia; ^3^ Harold and Margaret Milliken Hatch Laboratory of NeuroendocrinologyThe Rockefeller UniversityNew YorkUSA; ^4^ Centre for Integrative PhysiologyDeanery of Biomedical SciencesThe University of EdinburghGeorge SquareEdinburghUK

**Keywords:** Amygdala, dendritic spines, excitatory synaptic currents, synaptic plasticity

## Abstract

There is growing evidence that stress leads to contrasting patterns of structural plasticity in the hippocampus and amygdala, two brain areas implicated in the cognitive and affective symptoms of stress‐related psychiatric disorders. Acute stress has been shown to trigger a delayed increase in the density of dendritic spines in the basolateral amygdala (BLA) of rodents. However, the physiological correlates of this delayed spinogenesis in the BLA remain unexplored. Furthermore, NMDA receptors (NMDARs) have been known to underlie chronic stress‐induced structural plasticity in the hippocampus, but nothing is known about the role of these receptors in the delayed spinogenesis, and its physiological consequences, in the BLA following acute stress. Here, using whole‐cell recordings in rat brain slices, we find that a single exposure to 2‐h immobilization stress enhances the frequency, but not amplitude, of miniature excitatory postsynaptic currents (mEPSCs) recorded from principal neurons in the BLA 10 days later. This was also accompanied by faster use‐dependent block of NMDA receptor currents during repeated stimulation of thalamic inputs to the BLA, which is indicative of higher presynaptic release probability at these inputs 10 days later. Furthermore, targeted in vivo infusion of the NMDAR‐antagonist APV into the BLA during the acute stress prevents the increase in mEPSC frequency and spine density 10 days later. Together, these results identify a role for NMDARs during acute stress in both the physiological and morphological strengthening of synaptic connectivity in the BLA in a delayed fashion. These findings also raise the possibility that activation of NMDA receptors *during* stress may serve as a common molecular mechanism despite the divergent patterns of plasticity that eventually emerge *after* stress in the amygdala and hippocampus.

## Introduction

Accumulating evidence from animal models indicates that stress‐induced plasticity varies across different brain regions (McEwen and Sapolsky 1995; Popoli et al. 2012; Chattarji et al. 2015). For instance, earlier studies reveal that chronic stress causes dendritic atrophy in CA3 pyramidal neurons of the rodent hippocampus (McEwen 1999; Vyas et al. 2002). In the basolateral amygdala (BLA), by contrast, chronic stress triggers the opposite effect by strengthening the structural basis of synaptic connectivity through dendritic growth and spinogenesis (Vyas et al. 2002; Mitra et al. 2005; Roozendaal et al. 2009). Furthermore, the unique features of stress‐induced morphological plasticity in the BLA are not limited to chronic stress alone. The temporal profile of structural plasticity in the BLA can also be modulated by the duration of the stressor. For example, a single 2‐h exposure to immobilization stress leads to a significant increase in spine density that is evident only 10 days after the stress and not 1 day later (Mitra et al. 2005). Together, these studies have helped identify unique spatiotemporal features of stress‐induced plasticity in the amygdala that are quite distinct from those observed in the hippocampus.

However, compared to the decades of research on stress‐induced plasticity in the hippocampus, relatively little is known about the molecular mechanisms underlying the effects of stress in the amygdala. In the hippocampus, both stress and glucocorticoid treatment cause enhanced expression of the N‐methyl‐D‐aspartate (NMDA) subtype of glutamate receptors (Bartanusz et al. 1995; Weiland et al. 1997). Furthermore, pharmacological antagonism of NMDA receptors (NMDARs) blocks stress‐induced plasticity in the hippocampus and medial prefrontal cortex (mPFC) (Armanini et al. 1990; Magariños and McEwen 1995; Gould et al. 1997; Martin and Wellman 2011). However, as mentioned earlier, the patterns of stress‐induced morphological changes in the hippocampus/mPFC are generally opposite to those seen in the BLA (e.g., loss vs. growth of dendrites and spines) (Vyas et al. 2002; Mitra et al. 2005; Pawlak et al. 2005; Chen et al. 2008). This raises the question whether the same NMDARs can mediate contrasting patterns of structural plasticity in the amygdala. Moreover, many of the earlier findings were drawn primarily from models of chronic or repeated stress (e.g., 10 days of immobilization stress or 21 days of restraint stress) and their impact on plasticity soon after the end of stress. Nothing is known about how a brief stressor leads to the delayed spinogenesis in the BLA (Mitra et al. 2005; Rao et al. 2012). Therefore, this study focused on this gap in knowledge by addressing two specific questions. First, we investigated the physiological correlates of the delayed formation of new dendritic spines in the BLA 10 days after a single episode of 2‐h immobilization stress. Second, we examined if NMDA receptor activity in the BLA *during* this acute stressor plays a role in the eventual spinogenesis, and its physiological consequences, 10 days later.

## Materials and Methods

### Animals

Male Wistar rats (55–70 days old, 330–400 g) were housed in a 14/10‐h light/dark schedule (lights on at 8 am) with ad libitum access to food and water at the National Centre for Biological Sciences, Bangalore, India. The Institutional Animal Ethics Committee approved all procedures related to animal maintenance and experimentation.

### Stress

Rats were subjected to a single, 2‐h episode of acute immobilization stress (Mitra et al. 2005) between 10 am and 12 pm in plastic immobilization bags in a room different from that used for housing. Rats had no access to food or water during this 2‐h period. However, they had access to air through a sufficiently large opening at the tip of the immobilization bags adjacent to the rat's nose. After stress, animals were returned to home cages and subjected to electrophysiological/morphological analysis 10 days after acute stress.

### Targeted pharmacological infusion of APV into the BLA

Prior to surgery, rats were anesthetized with intraperitoneal administration of a cocktail of ketamine (80 mg kg^−1^ of body weight) and xylazine (10 mg kg^−1^). Body temperature was maintained with a heating pad. Rats were surgically implanted with bilateral, chronic, intracranial stainless steel guide cannulae (7 mm long, 26 gauge, Plastic One) aimed 1 mm above the BLA (stereotaxic coordinates: 2.5 mm posterior to bregma, ±5 mm lateral to midline, and 6.5 mm ventral from the bregma level) (Paxinos and Watson 2005). Intraamygdala infusions were carried out using standard procedures as described earlier (Wilensky et al. 2006). Briefly, after postsurgical recovery (7–10 days), infusions were performed either in the rats' home cage or while the rats were stressed in rodent immobilization bags by inserting infusion cannulae (31 gauge) through the guide cannulae. This was connected through polyethylene tubing to a Hamilton syringe (10 μL), which was mounted on an infusion pump (Harvard Apparatus, Holliston, MA). Rats were infused bilaterally with either vehicle (aCSF, 0.5 μL per side) or APV (0.5 μL per side, 1 μg μL^−1^ in aCSF) at a rate of 0.1 μL min^−1^. After waiting for 5 min, the infusion cannulae were taken out to allow the drug to diffuse.

### Preparation of amydalar slices and electrophysiology

Rats were deeply anaesthetized with halothane and decapitated. The brain was removed rapidly and transferred to ice‐cold artificial cerebrospinal fluid (aCSF: 115 mmol L^−1^ NaCl, 25 mmol L^−1^ glucose, 25.5 mmol L^−1^ NaHCO_3_, 1.05 mmol L^−1^ NaH_2_PO_4_, 3.3 mmol L^−1^ KCl, 2 mmol L^−1^ CaCl_2_, and 1 mmol L^−1^ MgCl_2_; (pH 7.4, 320 mOsm) and whole‐brain coronal sections (400 μm in thickness) containing the amygdala were cut using Vibratome 1000 Plus tissue slicer (Vibratome, St. Louis, MO) and then transferred to a storage chamber containing aCSF (room temperature; equilibrated with 95% O2/5% CO2), where they were allowed to recover for at least 1 h before being transferred to a submerged recording chamber at room temperature, attached to an Olympus BX51WI microscope.

Whole‐cell patch‐clamp recordings, from excitatory principal neurons in the BLA were obtained under IR‐DIC visualization (BX51WI, Olympus, Melville, NY). To this end, we selected neurons possessing large somata, which are typical of spiny excitatory principal neurons in the BLA (Mahanty and Sah 1999; Weisskopf et al. 1999). Whole‐cell pipettes (3–5 MΩ) were filled with (in mmol L^−1^): CsOH, 110; D‐gluconic acid, 110; CsCl, 10; HEPES, 10; NaCl, 8; QX‐314, 5; phosphocreatine, 10; Mg‐ATP, 4; Na‐GTP, 0.3; EGTA 0.2 (pH 7.3; 285 mOsm). Recordings were done using a HEKA EPC10 Plus amplifier (Heka Electronik) filtered at 2.9 kHz and digitized at 10 kHz. Monosynaptic EPSCs were elicited by stimulation of thalamic afferent fibers with a bipolar twisted platinum/iridium wire (2 × 25 μm, FHC, Bowdoin). Only cells with membrane potentials more negative than −60 mV were included in this study. In addition, series resistance (*R*_s_) was tested and recordings were not used if they changed by more than 20% from beginning to end for mEPSC recordings and 25% for MK‐801 recordings or if the *R*_s_ exceeded 25 MΩ.

#### mEPSC recording

Cells were held at −70 mV for recording miniature EPSCs (mEPSCs), which were pharmacologically isolated by adding TTX (1 μmol L^−1^) and picrotoxin (75 μmol L^−1^) to the recording solution. Continuous current traces of 5‐min duration (recorded at least 5 min after achieving whole‐cell configuration) were analyzed using the Mini Analysis Program (Synaptosoft, Fort Lee, NJ).

#### MK‐801‐induced decays of NMDAR currents

To assess changes in presynaptic release probability, NMDAR‐mediated component of evoked thalamic EPSCs was isolated by recording at a holding potential of +40 mV in the presence of picrotoxin (75 μmol L^−1^) and CNQX (10 μmol L^−1^). EPSCs were evoked every 20 sec. MK‐801 (5 μmol L^−1^) was added to the perfusate and stimulation was paused for 10 min to allow for equilibration. Stimulation was then resumed and the decay of NMDAR‐EPSC amplitudes measured. EPSC amplitudes were measured and normalized to the first trace in MK‐801. The time course the MK‐801 induced block of the subsequent 100 EPSCs was fitted by a single exponential in order to obtain a time constant that gave a measure of the number of stimuli required to produce an e‐fold decay of the NMDAR‐mediated EPSC amplitude.

Values are reported as mean ± SEM. Statistical comparisons were made using ‘*n*’ as number of cells after using Levene's test and single‐sample Kolmogorov–Smirnov (K–S) test for appropriate assumptions of variance and normality of distribution. Comparisons between two groups were done using unpaired Student's *t*‐test. Multiple‐group comparisons were carried out using ANOVA followed by post hoc pairwise tests. Comparison for cumulative distributions between two groups was done using two‐sample K–S test and Kruskal–Wallis test followed by post hoc pairwise tests were used for comparisons between multiple groups.

### Golgi staining and dendritic spine density analysis

Animals were sacrificed under deep halothane anesthesia. The brains were rapidly dissected out and placed in freshly prepared Golgi‐Cox fixative (5% potassium dichromate, 5% mercuric chloride, and 4% potassium chromate; 100 mL of each; 400 mL of water added later to make up the working fixative). After 8–10 weeks of incubation period, 120‐μm‐thick coronal sections of the brain were cut using a VibratomeVT1200 S (Leica). These sections were then developed using 5% sodium carbonate for 20 min and then dehydrated using a graded series of alcohol solutions, cleared in xylene, and mounted with DPX.

Coronal sections (120 μm) containing BLA (Bregma 1.92 to −3.12 mm) were used to visualize medium spiny pyramidal neurons (Paxinos and Watson 2005). An experimenter blind to treatment conditions quantified spine density. All protrusions (irrespective of morphological characteristics) on primary dendrite were counted as spines along an 80‐μm stretch using NeuroLucida (MicroBrightField Inc., Williston, Vermont) attached to an Olympus BX61 microscope (100X, 1.3 numerical aperture). Values are reported as mean ± SEM. Statistical significance was calculated using ANOVA followed by post hoc pairwise tests and n refers to the number of dendrites used for spine density analysis.

#### Chemicals

APV and MK‐801 was obtained from Tocris Bioscience (Avonmouth, Bristol, UK) or Sigma‐Aldrich (St. Louis, MO); TTX and QX‐314 was obtained from Alomone Labs, (Jerusalem, Israel). All other drugs were from Sigma‐Aldrich.

## Results

### Acute stress caused a delayed increase in the frequency of miniature excitatory postsynaptic currents in BLA principal neurons

**Figure 1:**
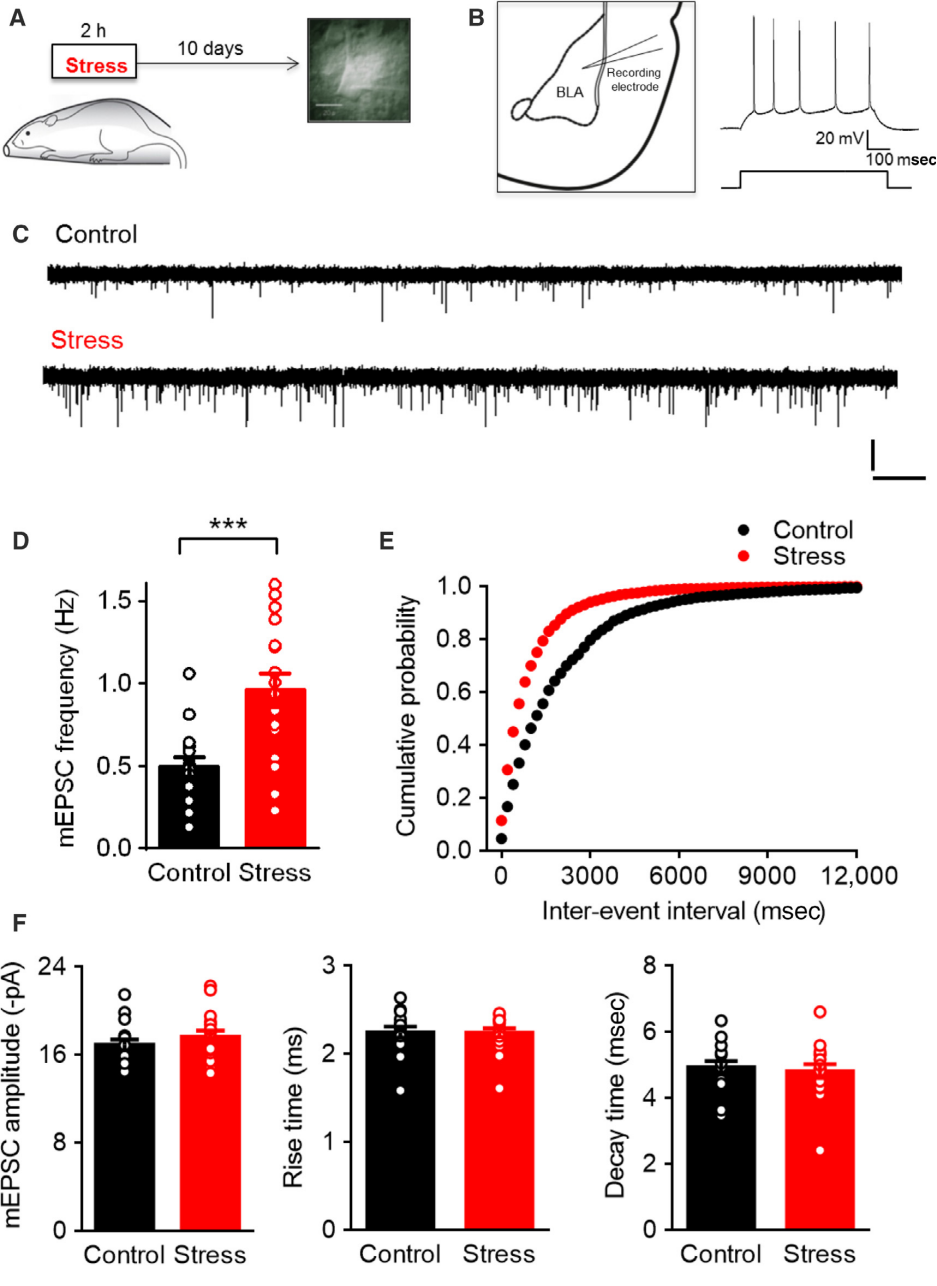
Acute stress causes a delayed increase in the frequency of miniature excitatory postsynaptic currents (mEPSCs) in BLA principal neurons 10 days later. (A) Schematic of experimental protocol: rats were subjected to 2 h of immobilization and 10 days later, animals were sacrificed for slice electrophysiology. (B) (*left*) Placement of recording electrode in a coronal slice of the amygdala. (r*ight*) Current‐clamp recordings of accommodating action potential firing (*top*) from a typical BLA principal neuron in response to depolarizing current injection (*bottom*). (C) Sample mEPSC traces from control and stress groups. (Scale bar: 20 pA, 2 sec). (D) Summary graph showing significant increase in frequency of mEPSCs in stress neurons compared with controls; Unpaired *t*‐test, ***P < 0.001. (E) Cumulative distribution plots of interevent interval for all events show a leftward shift with stress (K–S test, *P* < 0.0001). (F) No change was observed in mEPSC amplitude, rise time, or decay time with stress.

We previously reported that a single episode of 2‐h immobilization leads to a delayed increase in spine density on BLA principal neurons 10 days later (Mitra et al. 2005; Rao et al. 2012). As dendritic spines are the site of excitatory synaptic transmission in the mammalian brain, we first examined if this delayed strengthening in the structural basis of synaptic connectivity in the BLA also has a physiological correlate that is manifested as a delayed modulation of excitatory synaptic transmission. To this end, we used whole‐cell voltage‐clamp recordings in brain slices (Materials and Methods) taken from control and stressed rats to compare miniature AMPAR‐mediated excitatory postsynaptic currents (mEPSCs) in BLA principal neurons (Fig. 1A–C). Ten days after the 2‐h acute stress, BLA neurons from stressed animals exhibited a significant increase in mEPSC frequency (Control: 0.49 ± 0.06 Hz, *n* = 16; Stress: 0.96 ± 0.10 Hz, *n* = 17; Unpaired *t*‐test, ***P < 0.001; Fig. 1D), but not mEPSC amplitude (Control: 16.92 ± 0.48 pA, *n* = 16; Stress: 17.67 ± 0.53 pA, *n* = 17; Fig. 1F). This selective increase in mEPSC frequency was also reflected in a significant leftward shift in the cumulative frequency distribution toward shorter interevent intervals in stressed neurons [K–S test, *P* < 0.0001, Fig. 1E]. Stress had no effects on the rise time and decay time of mEPSCs (Fig. 1F).

### Acute stress caused a delayed increase in presynaptic release probability at the thalamic inputs to BLA neurons

**Figure 2:**
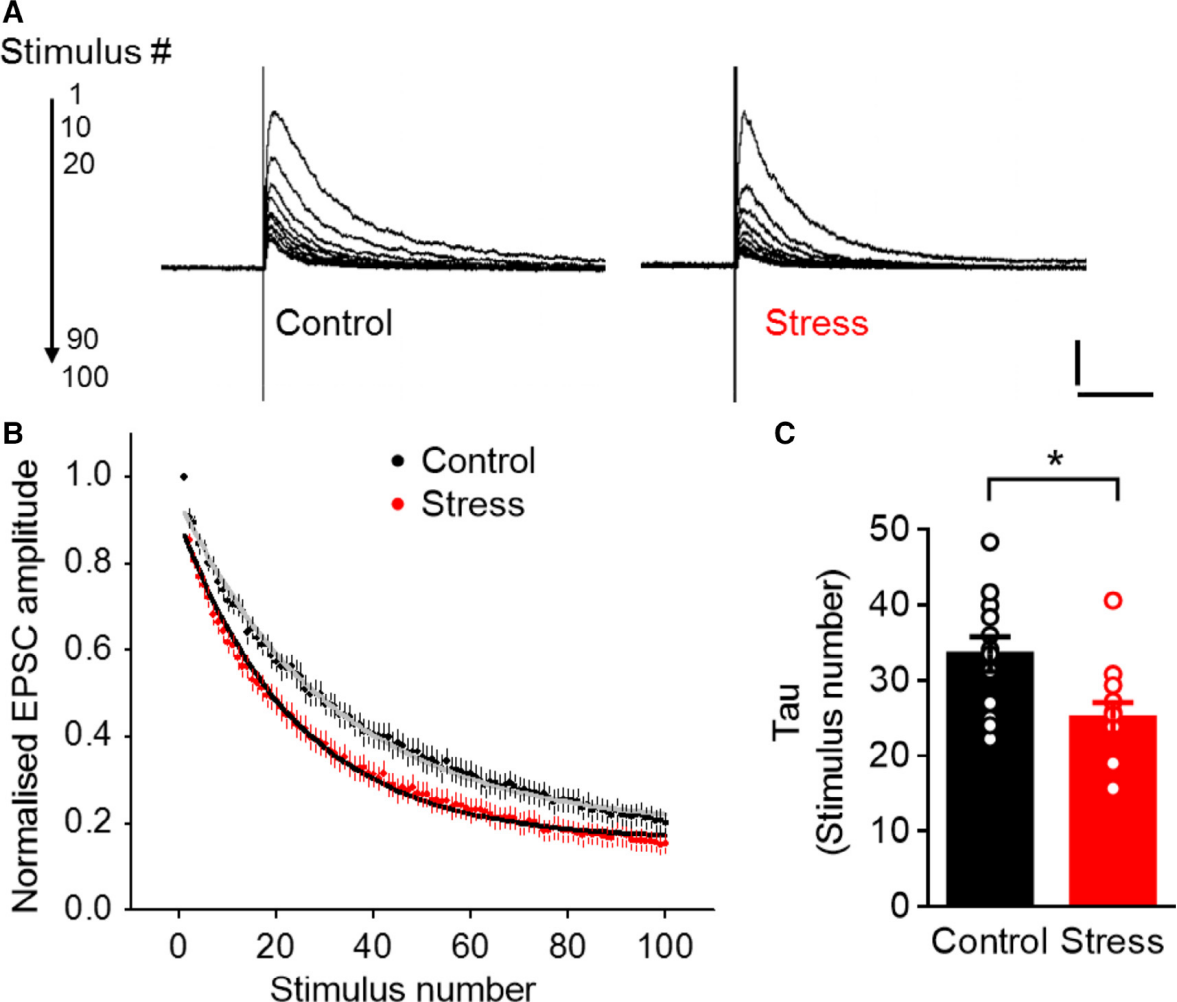
Acute stress causes a delayed increase in presynaptic release probability at the thalamic inputs to BLA neurons. (A) Representative traces illustrating the decay of NMDAR‐mediated EPSCs in the presence of MK‐801 in control and stressed animals with successive stimulus (Scale bar: 200 pA, 250 msec). (B) Summary of normalized mean amplitudes of NMDAR EPSCs evoked in neurons from control and stress groups by repeated stimulation of the thalamic fibers in presence of MK‐801. Stress results in a faster exponential decay compared to controls. (C) Average of decay constants derived from best exponential fits of NMDA‐EPSC decays shows a significant reduction with stress (Unpaired *t*‐test *P < 0.05).

While the stress‐induced enhancement in mEPSC frequency is consistent with an increase in the number of dendritic spines (Turrigiano and Nelson 2004), such an increase could also be caused by enhanced presynaptic transmitter release (Prange and Murphy 1999; Turrigiano and Nelson 2004). Therefore, we analyzed the potential effect of acute stress on presynaptic release probability (Pr) by repeated stimulation of the thalamic inputs to BLA cells in the presence of the NMDAR open‐channel blocker (5S,10R)‐(+)‐5‐methyl‐10,11‐dihydro‐5H‐dibenzo[a,d]cyclohepten‐5,10‐iminemaleate (MK‐801) (Fourcaudot et al. 2008). This led to a progressive decay of NMDAR‐EPSCs (Fig. 2A), the decay constant of which is inversely related to the probability of release (Rosenmund et al. 1993; Manabe and Nicoll 1994). The decay kinetics were fit to a single exponential and the time constant of decay was compared across slices taken from stressed and control animals (Fig. 2B). Ten days after acute stress, BLA neurons from stressed animals exhibited a significant reduction in the decay constant (Control: 33.47 ± 2.3, *n* = 12; Stress: 25.03 ± 2.03, *n* = 12; Unpaired *t*‐test *P < 0.05; Fig. 2C). These results point to an increase in Pr caused by the same acute stress that also enhances mEPSC frequency 10 days later.

### Targeted infusion of APV into the BLA during acute stress blocked the delayed increase in mEPSC frequency

**Figure 3:**
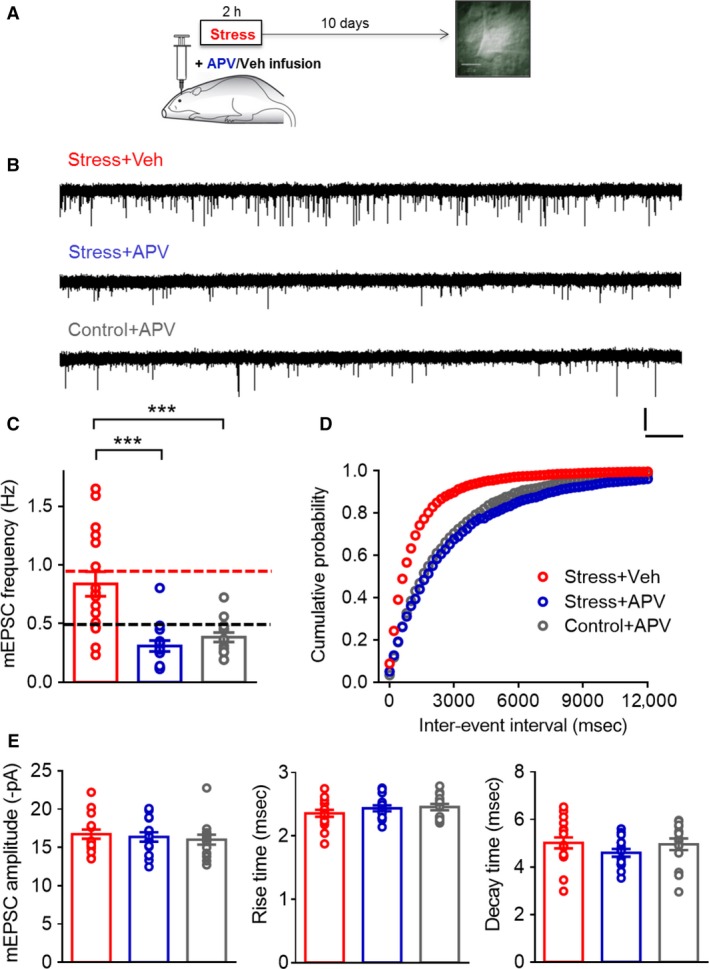
Targeted infusion of APV into the BLA during acute stress blocks the delayed increase in mEPSC frequency. (A) Schematic of experimental protocol: rats receive targeted, bilateral infusion of either vehicle (Veh) or the NMDAR‐antagonist 2‐amino‐5‐phosphono‐valeric acid (APV) into the BLA while being subjected to 2 h of immobilization, and 10 days later, animals were sacrificed for slice electrophysiology. (B) Sample mEPSC traces (Scale bar: 20 pA, 2 sec). (C) Summary of results: mEPSC frequencies are not different between vehicle‐infused stressed rats and intact rats following stress exposure (red dotted line). APV infusion prevents this stress‐induced increase. In fact, APV‐infused stress rats have mEPSC frequency values similar to control rats (black dotted line). Asterisk indicates significant differences (***P <* *0.001, One‐way ANOVA, Holm‐Sidak's multiple comparisons test). (D) Cumulative distribution plots of interevent interval for all events shows a leftward shift with stress, which is reversed by APV infusion during stress. (Kruskal–Wallis test, *P* < 0.0001; Dunn's multiple comparison; Stress + Veh vs. Stress + APV,* P* < 0.0001; Stress + Veh vs. Control + APV,* P* < 0.0001; Stress + APV vs. Control + APV, ns) (E) Summary of average mEPSC amplitudes, rise times, and decay times.

Next, we investigated the potential involvement of NMDA receptors in the BLA during acute stress in the delayed enhancement of mEPSC frequency 10 days later. To this end, rats received a targeted, bilateral infusion (Materials and Methods) of either vehicle (Veh) or the NMDAR‐antagonist 2‐amino‐5‐phosphono‐valeric acid (APV) into the BLA at the beginning of the 2‐h immobilization stress protocol (Fig. 3A). Ten days later, mEPSCs were recorded from BLA principal neurons in brain slices obtained from these animals (Fig. 3A and B, *top and middle*). We first confirmed that targeted in vivo infusion of vehicle into the BLA had no impact on the efficacy of acute stress in increasing mEPSC frequency in BLA neurons (Stress + Veh, Fig. 3C). The enhanced level of mEPSC frequency in stressed animals that received vehicle infusion into the BLA was not significantly different (unpaired *t*‐test, *P* = 0.96) from those seen earlier in intact animals after exposure to the same acute stress alone (the mean value for mEPSC frequency in stressed animals from Figure 1D are replotted as a dotted red line for comparison in Figure 3C). Strikingly, targeted infusion of APV into the BLA during the 2‐h stress prevented the stress‐induced enhancement in mEPSC frequency observed in Stress + Veh animals (the mean value for mEPSC frequency in control animals from Figure 1D is replotted as a dotted black line for comparison). This reversal of the stress‐induced enhancement in mEPSC frequency by APV was statistically significant (Fig. 3C, Stress + Veh: 0.84 ± 0.10 Hz, *n* = 17; Stress + APV: 0.30 ± 0.04 Hz, *n* = 15; One‐way ANOVA, *F*_(2,43)_ = 15.41, *P *<* *0.0001; ***P <* *0.001, Holm–Sidak's multiple comparisons test). Furthermore, the effects of APV infusion into the BLA were specific to stressed animals as it did not result in any additional reduction in mEPSC frequency in control animals (Control + APV: 0.38 ± 0.04 Hz, *n* = 14). The cumulative distribution of mEPSC interevent interval (Fig. 3D) in APV‐infused stressed animals showed a significant rightward shift relative to vehicle‐infused stressed animals but overlapped with control animals treated with APV (Kruskal–Wallis test, *P* < 0.0001; Dunn's multiple comparison; Stress + Veh vs. Stress + APV, *P* < 0.0001; Stress + Veh vs. Control + APV, *P* < 0.0001; Stress + APV vs. Control + APV, ns). Consistent with earlier results shown in Figure 1, mEPSC amplitude, rise time, or decay time was not affected by either vehicle or APV infusion into the BLA during the 2‐h stress (Fig. 3E).

### Targeted APV infusion into the BLA during acute stress also prevented the delayed increase in spine density

**Figure 4:**
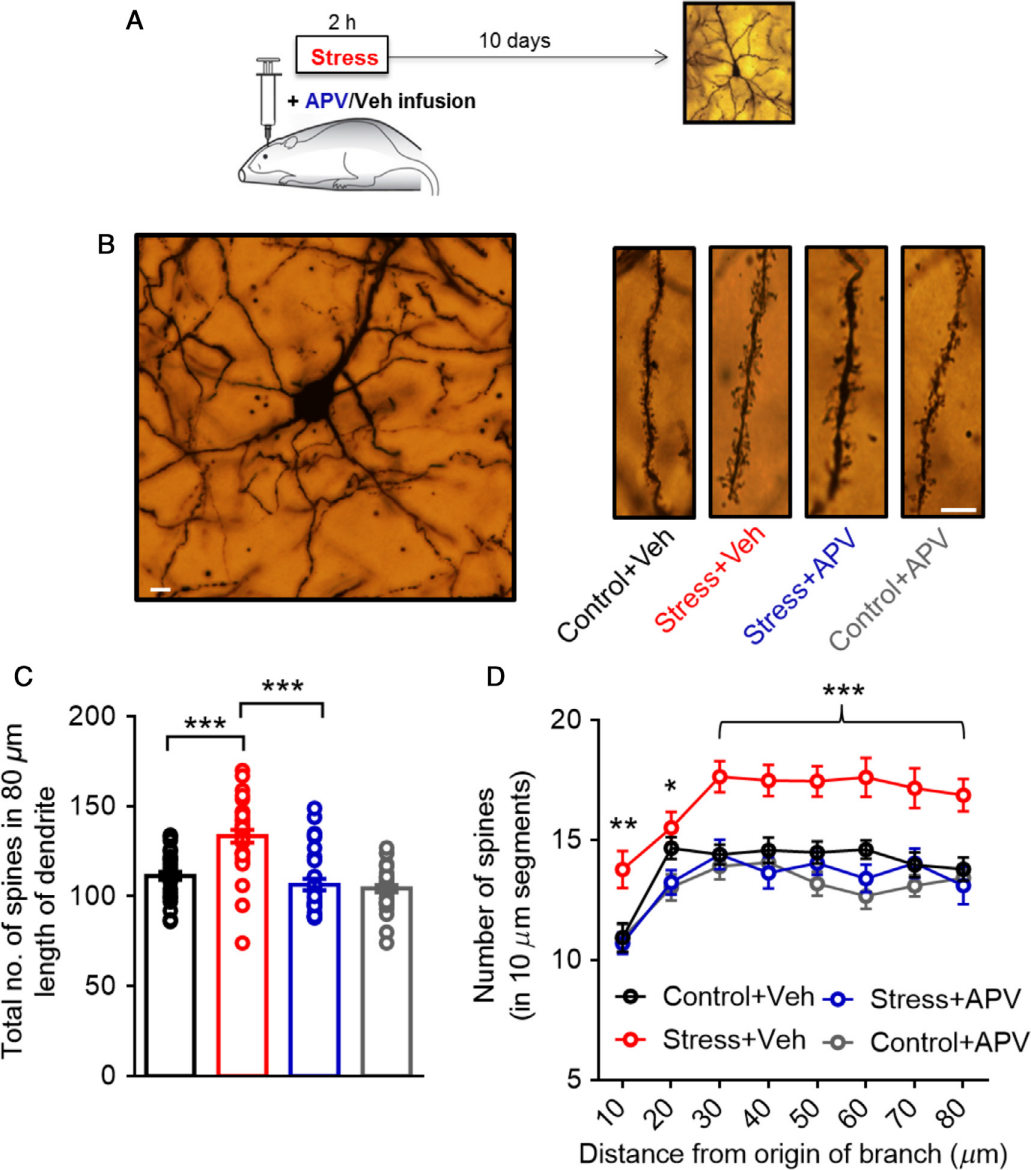
Targeted APV infusion into the BLA during acute stress prevents the delayed spinogenesis in the BLA. (A) Schematic of experimental protocol: rats receive targeted, bilateral infusion of either vehicle (Veh) or APV into the BLA while being subjected to 2 h of immobilization and 10 days later, animals were sacrificed for Golgi staining. (B) Low‐power photomicrograph of a Golgi stain‐impregnated pyramidal neuron in the BLA (20×, scale bar: 10 μm) (*left*). Representative images (*right*) of primary dendrites of BLA pyramidal neurons from control and stressed rats, under conditions of either Veh or APV infusion (40×, scale bar: 10 μm). (C) Acute stress causes a delayed increase in BLA spine density in vehicle‐infused animals 10 days later. APV infusion during stress leads to a significant decrease in the number of spines in the primary dendrites of the BLA compared to vehicle‐infused animals that were subjected to stress. Control animals infused with APV show levels comparable to stressed animals infused with APV. Asterisk indicates significant differences (***P <* *0.001, Two‐way ANOVA, Tukey's multiple comparisons test). (D) Segmental analysis of mean numbers of spines in each successive 10‐μm segment of the primary dendrite as a function of the distance of that segment from the origin of the branch. Significant differences observed in specific segments are indicated by * (for Stress + Veh vs. Stress + APV) as follows: *P < .05, **P < .01, ***P < .001 (RM two‐way ANOVA, Tukey's multiple comparisons test).

As described earlier, the acute stress paradigm used here has previously been shown to elicit a delayed increase in spine density in BLA principal neurons at the same10‐day time point (Mitra et al. 2005) when mEPSC frequency is enhanced. Therefore, the prevention of the physiological effects of acute stress by APV raises the possibility that blocking NMDA receptors could also reverse the morphological effects manifested as delayed spinogenesis in the BLA. To test this prediction, spine density on primary dendrites of BLA pyramidal neurons was quantified 10 days after acute stress in rats that were exposed to acute stress with infusions of either vehicle or APV into the BLA, as well as control rats that received the same APV or vehicle infusions, but without acute stress (Fig. 4A and B). Acute stress caused a delayed increase in BLA spine density in vehicle‐infused animals 10 days later (total number of spines, Control+Veh: 111.4 ± 2.2, *n* = 33; Stress + Veh: 133.5 ± 3.5, *n* = 31, Fig. 4B *right* and C). Notably, this stress‐induced spinogenesis was prevented by infusion of APV into the BLA (total number of spines, Stress + APV: 106.5 ± 3.3, *n* = 30; Control + APV: 104.2 ± 2, *n* = 33; Two‐way ANOVA, Factor stress: *F*_(1,123)_ = 18.89, *P* = 0.0001; Factor drug: *F*_(1,123)_ = 37.11, *P* = 0.0001; interaction: *F*_(1,123)_ = 12.53, *P* = 0.0006); ***P <* *0.001, Tukey's multiple comparisons test, Fig. 4B *right* and C). Furthermore, a more detailed segmental analysis of spine density along the dendritic length revealed how this prevention of delayed spinogenesis by APV infusion into the BLA is distributed across the length of primary apical dendrites analyzed (Fig. 4D). Interestingly, APV infusion into the BLA of control animals did not lead to further changes in spine numbers, suggesting that blocking NMDA receptors affects spine density only under stressed, but not normal, conditions (Fig. 4D).

## Discussion

This study is one of the first attempts to explore two key facets of the delayed spinogenesis in the basolateral amygdala (BLA) triggered by a single episode of acute stress – one in terms of its physiological correlates, and the other concerning a molecular mechanism. First, we tested whether a single 2‐h exposure to immobilization stress elicits changes in excitatory synaptic transmission that parallel the formation of dendritic spines reported previously in the BLA (Mitra et al. 2005). We find that the same stressor leads to a selective increase in the frequency, but not amplitude, of mEPSCs in BLA principal neurons at the same 10‐day time point after stress. In addition, this is accompanied by an increase in presynaptic release probability at the thalamic inputs to BLA neurons. Second, in light of earlier studies on the contribution of NMDA receptors to structural plasticity elicited in the hippocampus by chronic stress, we examined whether the same receptors also play a role in the physiological and morphological changes in BLA synaptic connectivity elicited by acute stress. Here, we report that, despite the contrasting features of stress‐induced plasticity in the amygdala compared to the hippocampus, not only does targeted in vivo pharmacological inactivation of NMDA receptors in the BLA during acute stress prevent its delayed impact on enhanced mEPSC frequency, but it also blocks the delayed increase in spine density 10 days later. Together, these findings identify a role for NMDA receptors during acute stress in both the physiological and morphological strengthening of synaptic connectivity in the BLA in a delayed fashion.

These results identifying a role for NMDA receptors in the BLA *during* acute stress in the eventual formation of dendritic spines are interesting for several reasons. Stress‐induced release of glucocorticoids has been shown to induce a rapid but transient increase in glutamate neurotransmission in the prefrontal cortex and hippocampus (Lowy et al. 1995; Karst et al. 2005; Yuen et al. 2009). The amygdala also shows a similar rapid increase in glutamate levels with stress; but unlike the hippocampus and prefrontal cortex, the increase in glutamate neurotransmission in the amygdala is longer lasting, for up to a few hours (Reznikov et al. 2007; Karst et al. 2010). This increase in glutamate levels could act upon NMDA receptors in the BLA to create an ideal synaptic substrate that eventually leads to a delayed formation of new dendritic spines. Furthermore, electrophysiological studies in amygdalar slices have demonstrated that in vitro application of stress levels of corticosterone leads to a reduction in GABAergic inhibitory synaptic transmission, as well as enhanced intrinsic excitability of BLA excitatory principal neurons (Duvarci and Paré 2007). Together, these factors could create conditions wherein stress‐induced disinhibition frees up the excitatory glutamatergic synapses on BLA neurons to undergo plasticity that eventually leads to a delayed strengthening of the structural basis of synaptic connectivity, manifested as newly formed dendritic spines in the BLA (Roozendaal et al. 2009).

How could NMDA receptors contribute to synaptic plasticity mechanisms that eventually culminate in the spinogenesis after acute stress? A significant body of evidence has demonstrated that formation of new dendritic protrusions and spines can be triggered by long‐term potentiation (LTP), a synaptic plasticity mechanism thought to underlie learning and memory (Engert and Bonhoeffer 1999; Yuste and Bonhoeffer 2001). Such LTP‐induced spinogenesis could be mediated through splitting of preexisting spines or spine formation de novo (Toni et al. 1999; Harris et al. 2003). Findings, derived mostly from studies in the hippocampus, suggest that NMDA receptors play a critical role in LTP and activity‐dependent spinogenesis (Fischer et al. 2000; Yuste and Bonhoeffer 2001). The existence of NMDA receptor‐dependent LTP in the BLA, and its role in fear memory, is also well established (Chapman et al. 1990; McKernan and Shinnick‐Gallagher 1997). While nothing is known about the delayed impact of a single episode of 2‐h immobilization stress on NMDA receptor currents, repeating the same stressor for 10 consecutive days (i.e., chronic immobilization stress) has been shown to enhance LTP and NMDAR currents by addition of NMDARs, not to preexisting AMPAR‐containing synapses, but to new synapses, thereby creating NMDAR‐only or so‐called “silent synapses” in the BLA (Suvrathan et al. 2014). Thus, in addition to the stress‐induced increase in glutamate levels that could act on NMDA receptors, stress could also modulate excitatory synaptic currents mediated by NMDA receptors. Also, the identity of these newly added receptors could itself be of consequence. Indeed, NMDA receptors containing NR2B subunits contribute to longer EPSPs, which provide longer time window for coincidence detection and hence affect synaptic plasticity (Monyer et al. 1994). Selective blockade of NR2B subunit by using ifenprodil not only impairs amygdalar LTP but also disrupts acquisition of fear learning (Rodrigues et al. 2001). Hence, our findings, pointing to a role for NMDA receptors in the delayed synaptic impact of an acute stress, raise the possibility that even a single episode of severe stress may leave its mark in the amygdala by generating new synapses with greater capacity for plasticity. Further experiments are necessary to explore if and how acute immobilization stress alters the extent or ability of BLA neurons to undergo LTP after stress. For example, it would be interesting to examine if there are specific time points when LTP may be affected, for example, during or soon after the end of stress or at later time points well after the end of stress.

What are the functional implications of the delayed electrophysiological effects of acute stress on mEPSCs alongside enhanced spine density? While the amplitude of mEPSCs is indicative of the strength of individual functional synapses, the frequency of mEPSCs is dependent on synapse number. Thus, the stress‐induced enhancement in mEPSC frequency is consistent with an increase in the number of dendritic spines (Burrone et al. 2002; Turrigiano and Nelson 2004). On the other hand, the absence of any change in the amplitude of mEPSCs suggests that the strength of individual synapses may not be affected by acute stress (Turrigiano et al. 1998). The increase in mEPSC frequency in BLA neurons in stressed animals also led us to examine potential changes in presynaptic release probability. To test this possibility, we compared probability of release (Pr) by repeatedly stimulating thalamic inputs to BLA principal neurons in the presence of the NMDAR open‐channel blocker MK‐801 (Rosenmund et al. 1993; Turrigiano and Nelson 2004; Fourcaudot et al. 2008). This analysis points to a delayed increase in Pr caused by the same acute stress that also enhances mEPSC frequency 10 days later. This provides novel evidence for a presynaptic enhancement in release probability concomitant with changes in spine number triggered by acute stress. Together these physiological (presynaptic release) and morphological (spine) changes may better account for the increase in mEPSC frequency seen 10 days after acute stress.

Finally, a role for NMDAR‐induced plasticity in the delayed effects of acute stress opens up new lines of investigation to test the efficacy of pharmacological interventions *after* stress. For example, how long after the end of stress would inactivation of NMDARs in the amygdala block the delayed effects on synaptic connectivity? Together, such studies will provide a useful framework for analyzing the gradual progression of the initial cellular and molecular changes in the amygdala triggered by a brief, severe stress that build up into amygdalar dysfunction and potential therapeutic interventions against it.

## Conflict of Interest

None declared.
